# Locomotion Mode Recognition for Walking on Three Terrains Based on sEMG of Lower Limb and Back Muscles

**DOI:** 10.3390/s21092933

**Published:** 2021-04-22

**Authors:** Hui Zhou, Dandan Yang, Zhengyi Li, Dao Zhou, Junfeng Gao, Jinan Guan

**Affiliations:** 1 School of Biomedical Engineering, South-Central University for Nationalities, Wuhan 430074, China; yddznmz@mail.scuec.edu.cn (D.Y.); 2012003@mail.scuec.edu.cn (Z.L.); zhoudao@mail.scuec.edu.cn (D.Z.); Junfengmst@163.com (J.G.); 3014507@mail.scuec.edu.cn (J.G.); 2 Key Laboratory of Cognitive Science, State Ethnic Affairs Commission, Wuhan 430074, China

**Keywords:** locomotion mode recognition, sEMG, ensemble learning, LightGBM

## Abstract

Gait phase detection on different terrains is an essential procedure for amputees with a lower limb assistive device to restore walking ability. In the present study, the intent recognition of gait events on three terrains based on sEMG was presented. The class separability and robustness of time, frequency, and time-frequency domain features of sEMG signals from five leg and back muscles were quantitatively evaluated by statistical analysis to select the best features set. Then, ensemble learning method that combines the outputs of multiple classifiers into a single fusion-produced output was implemented. The results obtained from data collected from four human participants revealed that the light gradient boosting machine (LightGBM) algorithm has an average accuracy of 93.1%, a macro-F1 score of 0.929, and a calculation time of prediction of 15 ms in discriminating 12 different gait phases on three terrains. This was better than traditional voting-based multiple classifier fusion methods. LightGBM is a perfect choice for gait phase detection on different terrains in daily life.

## 1. Introduction

Reliable powered lower limb exoskeletons and prostheses are of great significance to restore the gait of patients with lower limb disabilities. Intelligent prostheses are expected to provide assistance according to the user’s intent. The surface electromyography (sEMG) signal is the comprehensive effect of electromyography (EMG) and nerve stem electrical activity on the skin surface of shallow muscles, which can reflect the characteristics of human motion. The intent recognition of activities and motions based on sEMG has been widely used in the design of powered prostheses, and rehabilitation robots [1,2].

From existing research work, there are two applications of locomotion recognition based on sEMG: phase recognition and terrain recognition. The movement state of the lower limb during human walking is periodicity and repeatability. A gait cycle can be divided into eight events or periods at most: five events during stance phase and three events during the swing phase. These are, as follows: initial contact, loading response, midstance, terminal stance, preswing, initial swing, midswing, and terminal swing [3]. In some literature, load response and preswing events are ignored, and a gait cycle is divided into six phases. Joshi et al. used a linear discriminant analysis (LDA) classification algorithm to separate eight phases of gait using four channels of sEMG of the lower limbs, and the average accuracy was approximately 75% [4]. Peng discriminated six different gait phases with an average accuracy of 94.1% in a reasonable calculation time [5]. Some studies considered more realistic gait scenarios, and the gait phases were classified on different terrains [6], such as level-ground, ramp ascent/descent, and stair ascent/descent. Recently, researchers have focused their attention to gait events recognition during walking with cognitive tasks [7,8]. Referring to terrain recognition, the sEMG of the lower limbs during walking was acquired to identify five terrains [9]. Huang et al. developed an algorithm for terrain identification during walking [10], and seven movement modes were investigated: level-ground walking, stepping over an obstacle, ascending stairs, descending stairs, ipsilateral turning, contralateral turning, and standing still. In their study, one phase-dependent classifier was built for the sEMG of one gait phase window, in which at least one terrain decision could be made. The classification accuracy when using the phase-dependent classifier was higher than that when using one classifier trained by the EMG data from the entire stride cycle. In practical applications, phase recognition on a specific terrain or terrain recognition on a gait cycle is inadequate for prosthetic control. The prosthesis needs to be able to sense both the gait phase and terrain environment. Therefore, a locomotion recognition strategy is needed to classify phase events on different terrains that exist in daily life. Lower limb exoskeletons and prostheses can provide assistance, according to both gait phase and terrain information.

The feature extraction and selection method is an important issue in achieving the optimal classification performance in deploying sEMG as a control signal. Many extraction techniques, including the time domain features, the frequency domain features, the time-frequency domain features, auto-regression coefficients, and nonlinear features, have been proposed in the last two decades. Due to the low complexity of extraction methods without requiring signal transformation, time domain features and auto-regression coefficients have been used in motion recognition, with focus on real-time performance [11,12,13]. However, human motion and the sEMG data have been shown to be non-stationary in nature. Furthermore, the information of time domain features have weak robustness, and time-frequency domain features have been shown to be a more efficient tool to extract useful information from the sEMG signal. Among these techniques, EMG features based on wavelet analysis have been widely used in applications of pattern classification [14,15,16]. A high dimensional feature vector yielded by wavelet analysis can cause an increase in the learning parameters of a classifier. Therefore, a method of reducing the dimensionality of the feature vector was proposed. Methods based on the time domain or frequency domain were used to extract a feature of the wavelet vector [17].

Another important point in EMG motion recognition is the classifier design. With the continuous innovation of pattern recognition theory, many methods of pattern recognition have been put forward, such as LDA, support vector machine (SVM), K nearest neighbor (KNN), decision tree (DT), naïve Bayes, artificial neural network (ANN), to name but a few. The application of pattern recognition to myoelectric control schemes has been demonstrated in previous studies by employing various classifiers [18,19,20,21]. Furthermore, classifier fusion methods that combine multiple classifiers had better accuracy, when compared to any single classifier. Different classifier strategies, such as majority voting and weighted combination, have been adopted to improve the classification performance of the traditional classifier [22,23].

The present study discusses the intent recognition of gait events on three terrains based on sEMG, with the expectation to determining its applicability in reliable powered lower limb assistive devices. Section 2 details the employed methodology in the study, which includes the feature extraction, feature selection, and ensemble learning methods of classification. Section 3 presents the evaluation of features and classifiers. Finally, discussion and conclusions are shown in Section 4 and Section 5.

## 2. Materials and Methods

### 2.1. Participants

Four male subjects were recruited by convenience sampling. They were 21.50 ± 0.58 years old, 172.5 ± 5.0 cm tall, and 65.75 ± 10.90 kg in weight. All subjects had no history of neurological disorders, and can walk unassisted in daily life. They provided a written informed consent prior to participating into the study. The experimental protocol was approved by the South-Central University for Nationalities (Wuhan, China).

### 2.2. Experimental Protocol

Each subject was instructed to walk on three terrains: (1) The subject walked back and forth a 5-m long section of level ground. (2) The subject walked up a 0.64-m high four-step staircase. Each step was 0.6 m wide, 0.37 m deep, and 0.16 m high. (3) The subject walked down the four-step staircase. A 2-m long and 0.64-m high movable platform board connected the staircase to provide area for the end and beginning of walking. On each terrain, the subject walked at the preferred walking speed, and 30 trials were conducted. The subject sat for five minutes to rest after finishing tasks on a terrain.

### 2.3. The sEMG Recordings

Most of the important muscles in the lower extremities were active at both heel strike and toe off events. These were the periods of deceleration and acceleration of the legs, when the body weight is transferred from one foot to the other. Previous studies have analyzed the map of muscle relationships from the 14 EMG signals of the locomotion system [24]. Table 1 shows the relationships between all 14 muscles in the muscle space.

According to the division of the four muscle spaces, three muscles from the first three spaces and two muscles from the fourth space were chosen for the present experiments. The monitored muscles were the tibialis anterior (TA), medial gastrocnemius (MG), rectus femoris (RF), vastus lateralis (VL), and erector spinalis (ES).

**Figure 1:**
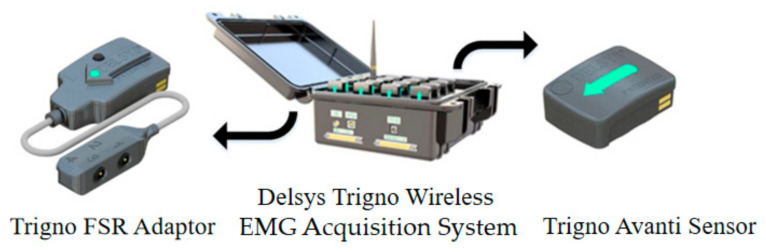
The wireless EMG acquisition system.

After careful preparation of the skin, the activity of five left lower limb and back muscles were recorded using wireless surface EMG electrodes (Delsys Trigno System, Natick, Massachuestts, USA). The location of each muscle belly was found using various anatomical landmarks for reference [25]. The EMG electrodes were secured to the body using double-sided tape and athletic bandage, in order to minimize the motion artifact. The data was filtered within 10–850 Hz, and sampled at 1926 Hz. During walking, the toe off (TO), toe strike (TS), heel off (HO), and heel strike (HS) events in a gait cycle were extracted through the foot pressure insole data sampled at 148 Hz (FSR Adapter, Delsys Trigno System). All data were transmitted to the EMG acquisition software on the computer by Wi-Fi (Figure 1).

### 2.4. Data Preprocessing

The raw EMG data was resampled to 500 Hz, bandpass filtered at 10–150 Hz, and notch filtered to remove 50 Hz and its harmonics components. Next, the wavelet denoising algorithm was used to subtract the motion artifact due to electrode movement during walking. The foot pressure data was also resampled to 500 Hz. Then, the TO and HS gait event timings were extracted. The consecutive EMG data on the three terrains were divided into gait cycles, according to the left heel strike event timings. A gait cycle occurred over the time between a HS event and consequent HS event. The TO event was relative to the beginning of the swing phase, and the HS event was relative to the beginning of the stance phase. During walking on the stair descent terrain, although the toe touched the staircase before the heel, the cycles were still picked based on the timing of the HS.

**Figure 2:**
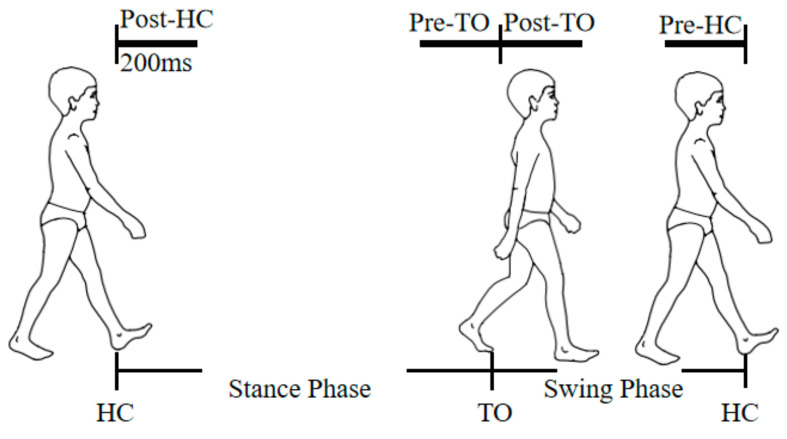
Four analysis windows aligned with heel contact (HC) and toe-off (TO).

After removing the EMG data during the gait cycle transition to and from rest, and data with poor quality, a subject completed 49 ± 18 cycles of ground walking, 41 ± 9 cycles of stair ascents, and 35 ± 2 cycles of stair descents on average. Pattern recognition was performed on four analysis windows aligned with the HS and TO gait events. These were, as follows: immediately after HC (Post-HC), prior to TO (Pre-TO), immediately after toe-off (Post-TO), and prior to the next HC (Pre-HC). For each analysis window, a set of features was calculated, and provided to the pattern classifier. One of the challenges in using EMG signals to classify a user’s movement pattern is that the recorded EMG signals are time-varying. The features of EMG signals from the leg and back muscles on analysis windows with short duration generally show large variations within the same gait mode, which might result in low performance for pattern recognition. A larger length segment of EMG has a stationary property, but imposes high computational load and a challenge to perform real-time prosthesis control. Real-time constraints enforce a delay time of less than 300 ms between the onset of muscle contraction made by a subject, and the corresponding motion in a device [26]. In the literature, less 256-ms transient EMG signals were used to obtain high movement classification accuracy [10]. These results imply a segment of EMG signals with a length of 256 ms, although time-varying, contain enough information to estimate a motion mode. Based on the study above, the duration of each analysis window was defined as 200 ms for the present study. In real-time motion control scenarios, enough time (at least 100 ms) can be left for features computation, classification, control commands generation, and prosthesis device response. The definition of four analysis windows in a gait cycle were shown in Figure 2. It is noteworthy that data overlapping was observed between the Post-TO and Pre-HC windows for the tasks of level-ground walking and descending stairs. For level-ground walking, subject #2, #3, #4 showed an maximum of 22, 1 and 40 ms of data overlapping separately; for the task of descending stairs, subject #3, had 16 data overlapping, ranging from 8 to 60 ms.

### 2.5. Data Analysis

#### 2.5.1. Feature Extraction

A feature is an individual measurable property of the process being observed [27]. Many feature extraction techniques have been proposed in several domains. Due to the implementation and computation simplicity, time domain features and auto-regression coefficients are the most popular in EMG movement recognition. In the present study, 12 features were preliminary selected, which included the mean absolute value (MAV), Willison amplitude (WAMP), number of zero crossings (ZC), waveform length (WL), number of slope sign changes (SSC), logarithm of variance (LogVAR), root mean square (RMS), 75% quantile (Q3), and the four coefficients of the fourth order auto-regressive model (AR1, AR2, AR3 and AR4). Furthermore, frequency domain features were commonly used to detect neuromuscular disease and muscle fatigue [28,29], and used for EMG hand movement recognition in few cases [30,31]. Five features in the frequency domain were evaluated. These were, as follows: median frequency (MDF), mean frequency (MNF), frequency ratio (PR), peak frequency (PKF), and mean power (MNP). Due to the non-stationary characteristic of human motion and EMG data, time-frequency domain features have been shown to be more representative of these types of data. Features based on discrete wavelet transform (DWT) have been widely adopted to extract useful information from the EMG signal [32,33]. The Daubechies (Db) wavelet family has been proven to be the most suitable wavelet for EMG signal analysis. However, the high dimensional feature vector yielded by DWT can lead to the increase in the classifier’s learning parameters. In order to reduce the dimensionality of the feature vector, the single feature statistic was calculated from the wavelet coefficient array. In the present study, the Db1, Db4 and Db7 wavelet bases were selected, and the decomposition level was set at 3. The DWT respectively generates the coefficient subsets at the third level approximation (cA3), and the first to the third level details (cD1, cD2 and cD3). In general, the low-frequency components (cA3 and cD3) of the EMG always contain an irrelevant low-resolution background, while the first and second decomposition levels (cD1 and cD2) are similar to the original EMG signal. Therefore, the investigators chose signals cD1 and cD2 to calculate the MAV, WAMP and WL features. Finally, 18 (3 wavelet bases ×2 decomposition levels ×3 features) time-frequency domain features were obtained. The MAV of the cD1 obtained through the Db1 wavelet was called Db1-cD1-MAV, and so on. The selection of wavelet bases, coefficient subsets and feature statistics were based on the results of existing literature [17,34,35].

#### 2.5.2. Feature Selection

Certain features can give good results in certain environmental conditions. The focus of the feature selection was to select a subset of property from the input, which can efficiently describe the input data, while reducing the effects of noise or irrelevant variables, and still providing good prediction results [36]. In general, there are two methods of feature selection: the measure of classification accuracy and the evaluation using statistical criteria. The first method evaluates EMG features through prediction results, depending on the classifier type. The second method aims to quantify the classification ability of the feature space. The present study focused on the second feature selection method. Three existing statistic criteria were used to evaluate the 35 EMG features above: the Davies-Bouldin index [37], scattering index [38], and rate between the Euclidean distance and standard deviation [39].

(1) Davies-Bouldin (DB) index
  The DB index is an evaluation metric for cluster separability, which is calculated, as follows:
  (1) $$DB=\frac{1}{L}\sum_{i=1}^{L}\underset{i\neq j}{\max}\frac{D_{ii}+D_{jj}}{D_{ij}}$$
  where: *L* is the total number of classes, and set to 12 (four phases in three terrains); *D_ii_* and *D_jj_* are the dispersions of the *i*th and *j*th classes, respectively; *D_ij_* is the Euclidean distance between the mean values.
  As observed from the formula: the DB index is the average of the worst situation distances of two by two clusters. A lower DB index implies a higher degree of cluster separability.
(2) Scattering index
  The scattering index considers the combination of all clusters to estimate the feature space quality. This is calculated, as follows:
  (2) $$J=\frac{\mathrm{trace}(S_{W})}{\mathrm{trace}(S_{B})}$$
  where: *S_W_* and *S_B_* are the covariance matrices of all classes and between-classes, respectively.
  It is obvious that the lower value of the scattering index signifies a better feature space quality.
(3) The rate between the Euclidean distance and standard deviation (*RES*)
  The *RES* index considers both separation index between classes and variation index in the same class. This can be expressed, as follows:
  (3) $$RES=\frac{1}{L(L-1)}\sum_{i,j=1}^{L}RES_{ij}=\frac{1}{L(L-1)}\sum_{i,j=1}^{L}\frac{D_{ij}}{\frac{1}{2}(\delta_{ii}+\delta_{jj})}$$
  where: *D_ij_* is the Euclidean distance between the mean values of the *i*th class and *j*th class; *δ_ii_* and *δ_jj_* are the standard deviations; *RES* is the average of *RES_ij_* from all possible combinations of *L* classes. A good classification performance can be obtained when the RES index is high.
  The DB index, scattering index and RES index of the 35 EMG features were calculated. For the convenience of comparison, the inverse of RES index (invRES) was obtained. Features with lower values for these three indexes had better class separability.
(4) Sensitivity to noise (*STN*)
  In addition to class separability, the sensitivity of feature space to environmental noise was considered to evaluate robustness. The standard deviation of the index among the four subjects was calculated to measure the robustness of the features. A lower standard deviation implies stronger robustness.
  (4) $$STN=\sqrt{\frac{\sum_{i=1}^{n}{\left(index_{i}-\bar{index}\right)}^{2}}{n-1}}$$
  where: $\bar{index}$ is the mean value of the *n* subjects; $index_{i}$ is the value of *i*-th subject; *n* is set to four.
  Features with good class separability and robustness were selected to form a feature vector, and these were provided to the classifier.

### 2.6. Classification

Two ensemble learning methods were considered in the present study: classifier fusion and light gradient boosting machine.

#### 2.6.1. Classifier Fusion

**Figure 3:**
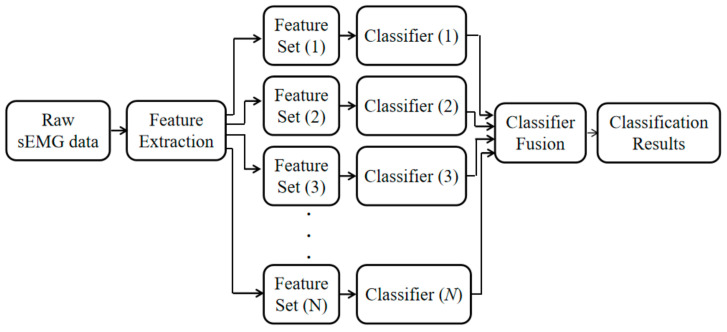
Flow diagram for the multiple classifier strategy.

A certain classifier fusion method can obtain a classification accuracy that is better than that can be obtained from individual classifiers. The flowchart for the multiple classifier strategy employed in the present study is shown in Figure 3.

The *N* features were extracted from the sEMG data. Then, individual classifiers were generated from each feature. The class labels generated by *N* classifiers were subsequently provided to the fusion method, in order to obtain a final classification result. The investigators set *N* as 6 in the present study.

In many classification methods, LDA has been proven to be highly efficient and unsusceptible to overfitting, and SVM has a high potential for recognizing patterns that are more complex. LDA and SVM classifiers have been applied to upper limb motion and gait phase classifications, in order to obtain the good classification results [7,20]. In the present study, the investigators applied both the LDA and SVM classifier to recognize lower limb motion from sEMG signals. Two kinds of fusion methodologies were investigated.

(1) Plurality majority voting fusion
  For majority voting fusion, a simple summation of class labels was used to decide the classification result. The equation is, as follows:
  (5) $$l_{i}=\underset{j=1}{\overset{N}{\max}}\sum_{i=1}^{L}l_{i},j$$
  where: *l* is the class label, *L* is the number of classes, and *N* is the number of classifiers. The result of the fusion method is the class label with the greatest number of votes in *L* class labels. If more than one label gets the most votes at the same time, one will be randomly selected.
(2) Weighted majority voting fusion
  In general, individual classifiers are not of identical accuracy when classifying a class. A classifier with higher accuracy in a class should have a greater say in the final class label. Hence, it is reasonable to weigh the classifiers. This can be implemented based on the following expression:
  (6) $$l_{i}=\overset{N}{\underset{j=1}{\mathrm{ma}}}x\sum_{i=1}^{L}b_{i}d_{i},j$$
  where: *d* is the individual result of a classifier, and *b* is the numerical weight assigned to this.

The *L*-dimensional quantity *w* was assigned to each classifier, which can be expressed as below:

(7) $$w=[b_{1}{}^{}b_{2}{}^{}b_{3}{}^{}\ldots b_{L}]$$

For *w*, *b_i_* (*1 ≤ i ≤ L*) represents the confidence of the classifier in predicting the *i*th class.

The *N* class labels from *N* classifiers were combined by the summation of the columns of the matrix shown below:

(8) $$W={\left[w_{1}{}^{}w_{2}{}^{}w_{3}{}^{}\ldots w_{N}\right]}^{T}={\left(b_{ij}\right)}_{N\times L}$$

After the summation of the columns, the *L* values were compared, and the maximum was designated as the final classification result.

#### 2.6.2. Light Gradient Boosting Machine

Light gradient boosting machine (LightGBM) is an effective parallel algorithm for the gradient boosting decision tree. LightGBM leverages the local statistical information contained in each machine through two-stage local voting and a global voting process, in order to identify the most informative attribute, and uses the full-grained histogram algorithm to speed up the arithmetic process. Experiments have shown LightGBM can achieve high accuracy at a very low communication cost [40].

#### 2.6.3. Training and Testing Model

The EMG feature data were segmented into the train data set and test data set. K-fold stratified cross-validation was used for the more precise estimation of classification performance. All available data were randomly partitioned into K subsets. Stratification means that the class abundance in the original feature data is maintained in each subset. Among these K subsets, a single subset was retained as the validation data for testing the model, and the remaining K−1 subsets were used as training data. Then, the cross-validation process was repeated for K times (folds), with each of the K subsets used exactly once as validation data. The K results from these folds can be averaged (or otherwise combined) to produce a single estimation. The stratified 5-fold cross-validation was used for the present study.

In the method of weighted majority voting fusion, a 5-fold cross-training, similar to the 5-fold cross-validation, was used to obtain the K-dimensional weights of each classifier. The K−1 subset training data was further partitioned into five subsets. The weights of the individual classifiers derived from the classification results of the training data were applied to validate the data for testing the model.

## 3. Results

### 3.1. The sEMG Data

**Figure 4:**
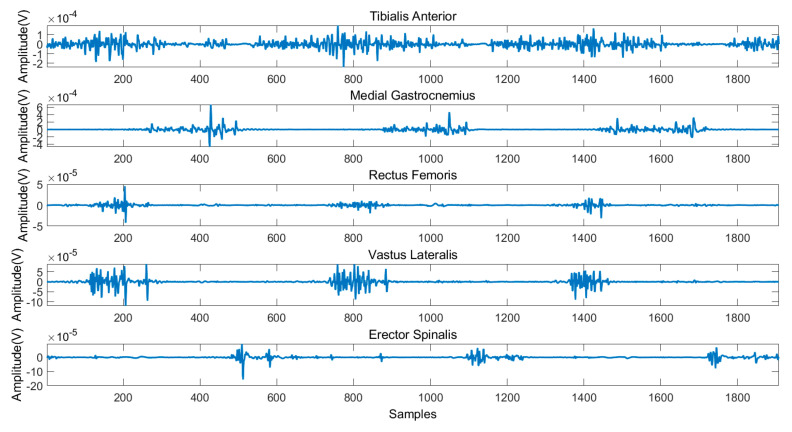
Typical sEMG signals during level ground walking.

**Figure 5:**
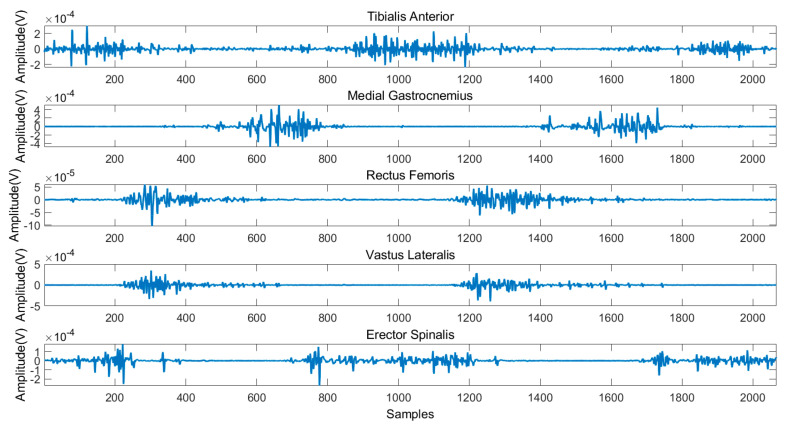
Typical sEMG signals during walking up the stairs.

**Figure 6:**
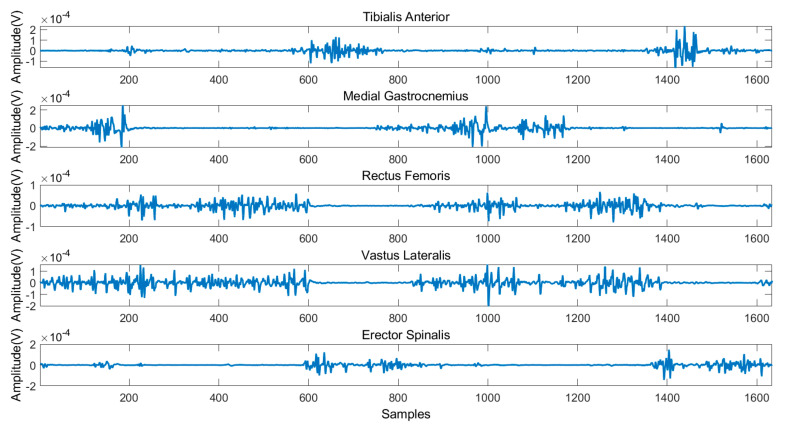
Typical sEMG signals during walking down the stairs.

Figure 4 illustrates the typical sEMG signals of three gait cycles during level ground walking. Figure 5 and Figure 6 illustrates the sEMG signals of two gait cycles during walking up and down the stairs, respectively. It can be observed from these figures that the data measured in three experimental scenarios had an obvious difference for the reason that the same muscle activates at different time points of a gait cycle.

### 3.2. Features Evaluation

**Figure 7:**
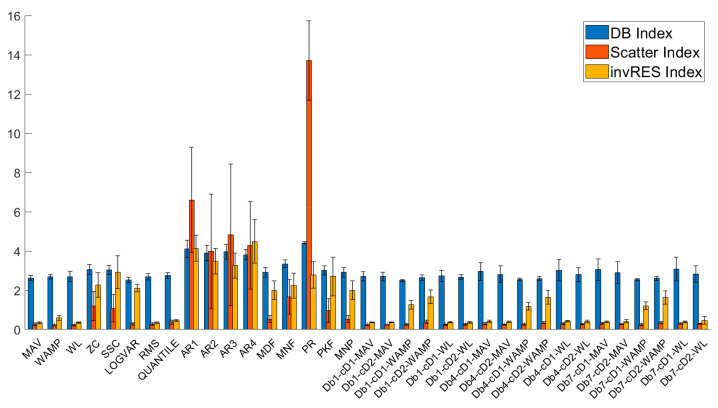
The separability index of features.

The class separability, index mean value, and standard deviation of the 35 EMG features are shown in Figure 7. The results show that all features have a similar DB Index. The ZC, SSC features of the time domain, AR1~AR4, and MNF, PR and PKF features of the frequency domain had higher values in both the Scattering and invRES indexes. The LogVAR, MDF, MNP and Db_N_-cD_M_-WAMP (N = 1, 4 and 7; M = 1 and 2) features of the time-frequency domain had higher values only in the invRES index. In addition, higher indexes were usually accompanied by higher standard deviations.

A similar DB index failed to distinguish the class separability of the features. Most features with a high invRES index also have high values in the scattering index. Furthermore, due to the lower complexity of computation and implementation, when compared to the other two methods, the invRES index can be a more effective indicator to evaluate the features.

Based on these above calculation results, three time-domain features and three time-frequency domain features were chosen as classifier inputs. These were, as follows: MAV, WL, RMS, Db_1_-cD_2_-MAV, Db_1_-cD_1_-WL, and Db_1_-cD_2_-WL.

### 3.3. Classification Results

The features were individually extracted from the data collected from the four subjects. Each of the six chosen features were ran on the LDA and SVM classifiers, in order to classify the twelve locomotion. Five types of ensemble learning methods were considered: majority voting fusion based on LDA (MV-LDA), majority voting fusion based on SVM (MV-SVM), weighted majority voting fusion based on LDA (WV-LDA), weighted majority voting fusion based on SVM (WV-SVM), and LightGBM.

#### 3.3.1. One Classifier

**Figure 8:**
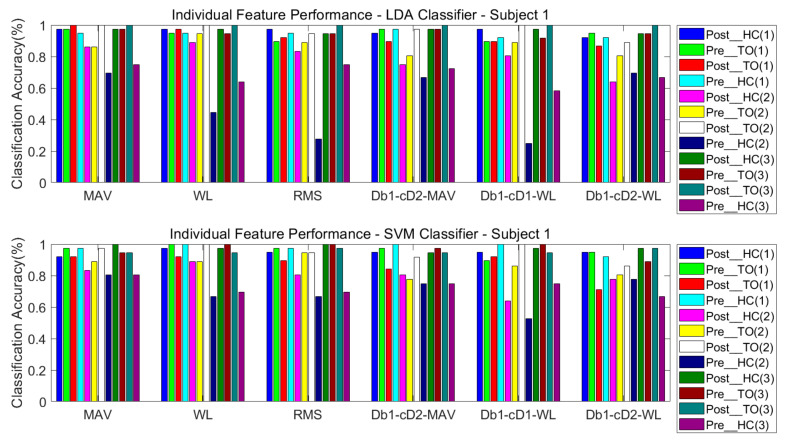
Classification performance of individual sEMG features; Subject #1.

**Figure 9:**
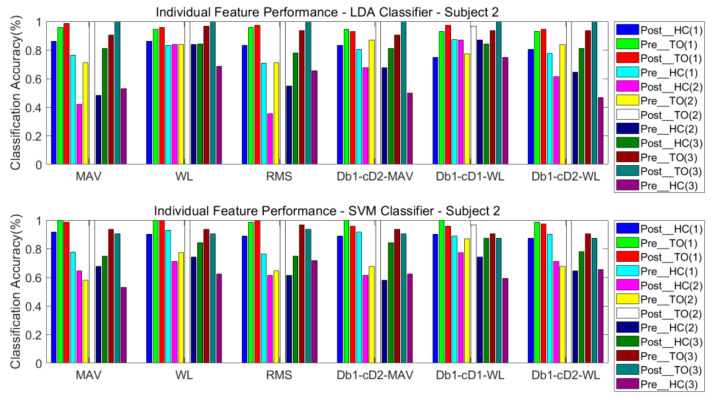
Classification performance of individual sEMG features; Subject #2.

**Figure 10:**
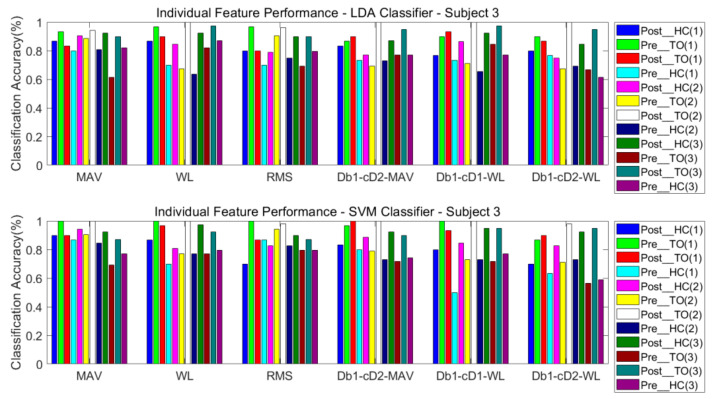
Classification performance of individual sEMG features; Subject #3.

**Figure 11:**
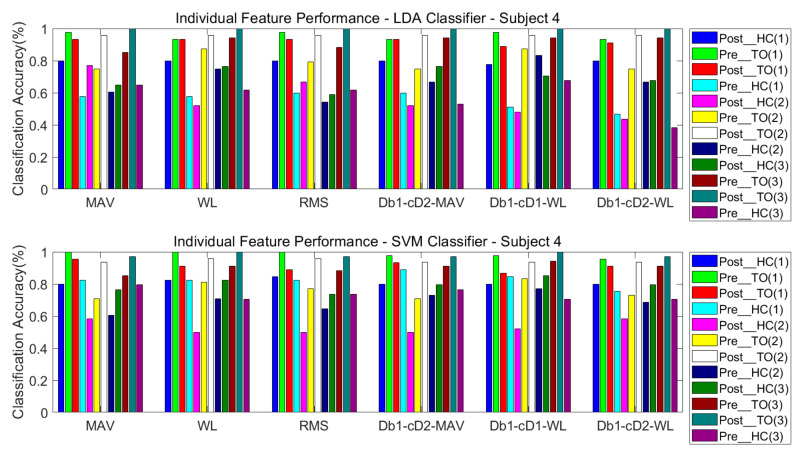
Classification performance of individual sEMG features; Subject #4.

The classification performance of six features for the four subjects is graphically shown in Figure 8, Figure 9, Figure 10 and Figure 11. The legends were placed on the right side of the figures. The number 1, 2 and 3 in the brackets refer to the level-ground, stair ascent, and stair descent terrains, respectively. In Figure 8, it can be observed that the classification accuracy of all features in the LDA classifier can reach up to 100% for Post_TO on the stair descent terrain. The worst available classification accuracy was 25% for Pre_HC on the stair ascent terrain using the Db_1_-cD_1_-WL feature for subject #1. In the SVM classifier, the classification accuracy for Post_TO on the stair ascent terrain was lower than 100%, but the classification accuracy for Pre_HC on the stair ascent terrain increased to over 52%. These results show that these classification accuracies are mutually exclusive, and that no single feature has an equal classification accuracy for all classes. A similar inference can be made from Figure 9, Figure 10 and Figure 11.

Table 2 and Table 3 show the overall classification accuracy for the twelve locomotion. It can be observed that LDA performs significantly weaker with the same feature in most cases. The SVM classifier can offer classification performance that matches or exceeds LDA classifier.

#### 3.3.2. Ensemble Learning

**Figure 12:**
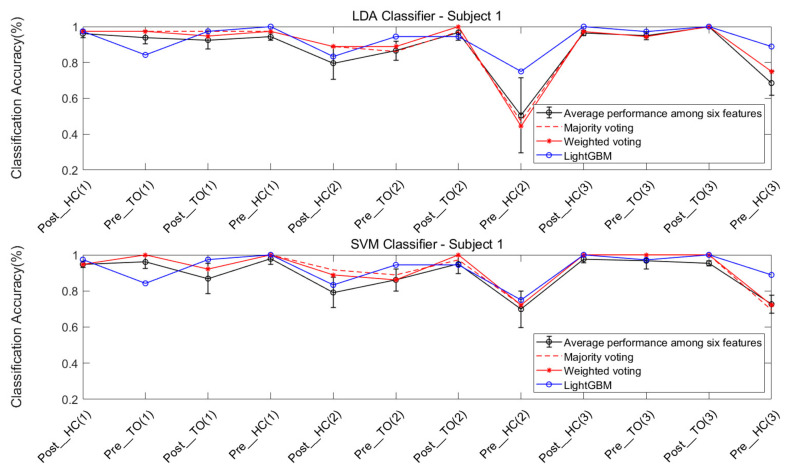
Classification performance for the ensemble learning method; Subject #1.

The classification performance of ensemble learning method for Subject #1 is shown in Figure 12. The classification accuracy based on the LDA classifier and SVM classifier can be observed in the two sub-graphs above and below the figure. For a better comparison of performance, a circled black line, a dashed red line, an asterisked red line, and a circled blue line were added, indicating the average performance of the six features, and the performance of the majority voting fusion, the weighted majority voting fusion, and LightGBM, respectively.

First, it can be observed that there was a more dramatic improvement for the SVM classifier, when compared to the LDA classifier (Figure 12). This result is consistent with the result in Figure 8.

Second, the improvement in classification accuracy for most locomotion can be observed in the majority voting fusion and weighted majority voting fusion methods, when compared to the average performance of the six features. The only exception is the Pre_HC on the stair ascent terrain in the LDA classifier. In Figure 8, this locomotion had the worst classification accuracy, regardless of the feature used. In this case, ensemble learning based on multiple classifiers may exhibit a poorer performance. The reason is that multiple classifiers fuse the classification results of an individual classifier. If the latter yields a similar classification performance, the voting fusion would allow good to be better and bad to be worse.

Third, the majority voting fusion method and weighted majority voting fusion method are close in terms of classification performance. The reason is that the weights of individual classifiers in the weighted majority voting fusion method were derived from the classification results of the training data. Due to the similar classification performance of the individual classifier in the training data, the individual classifier would have a similar weight vector. In order to obtain better results, the multiple classifiers based method should guarantee a certain classification accuracy of the individual classifier, and also allow for a high degree of classification performance independence under various locomotion.

Finally, an improvement in the classification accuracy of most locomotion can be observed in LightGBM, when compared to the other three results, regardless of the classifier. Although the Pre_HC on level-ground, and the Post_HC and Post_TO on the stair ascent terrain had lower classification accuracies in LightGBM, when compared to those in the two voting fusion methods, an improvement in the classification accuracy of Pre_HC on the stair ascent terrain can be acquired in LightGBM. It can be observed that the classification accuracy for this locomotion improved from 44.44% to 75.00%, when compared to the methods in the LDA classifier, improved from 72.22% to 75.00%, when compared to methods in the SVM classifier.

**Figure 13:**
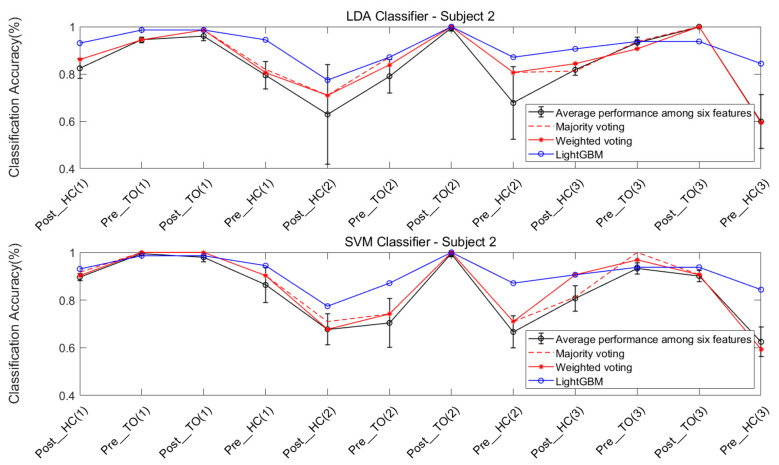
Classification performance for the ensemble learning method; Subject #2.

**Figure 14:**
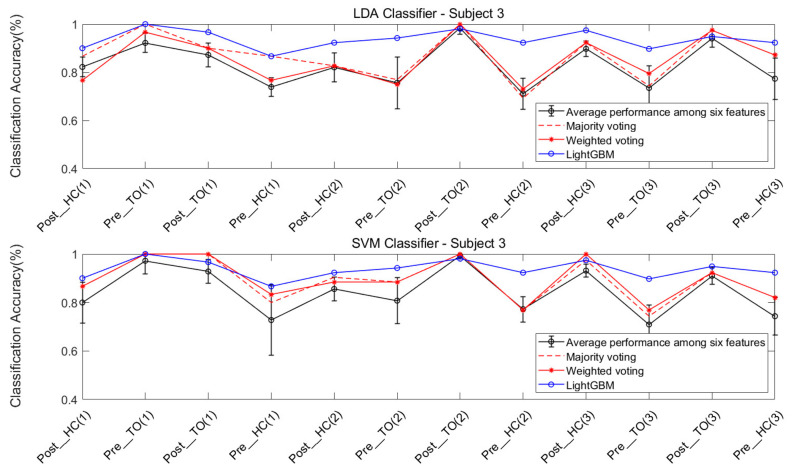
Classification performance for the ensemble learning method; Subject #3.

**Figure 15:**
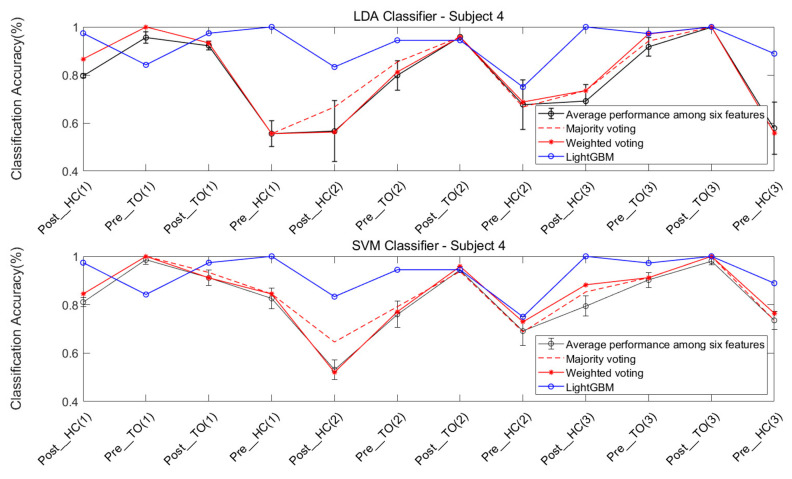
Classification performance for the ensemble learning method; Subject #4.

Similarly, Figure 13, Figure 14 and Figure 15 show the classification performance of the ensemble learning method for Subject #2–#4, respectively. The same results shown in Figure 12 can be extended to Figure 13, Figure 14 and Figure 15. In Figure 13, a very dramatic improvement in the classification accuracy for Pre-HC on the stair descent terrain can be observed in LightGBM (from 59.38% to 84.36%), when compared to the other methods, and there was a considerable improvement in the classification accuracy of Post_HC on the stair ascent terrain (from 67.74% to 77.42%) and the Pre_HC on the stair ascent terrain (from 70.97% to 87.1%) for Subject #2. In Figure 14 and Figure 15, it was also revealed that LightGBM had an improved classification performance for Subject #3 and #4.

Table 4, Table 5, Table 6 and Table 7 illustrate the detailed results of the five ensemble learning methods. The average classification accuracy for LightGBM among four subjects was 93.1%.

The *macro-F1* score of the five models were calculated to measure the generalization performance of five classification algorithms. The definition of the *macro-F1* score is given, as follows:

(9) $$macro-F1=\frac{2\times \frac{1}{L}\sum_{i=1}^{L}P_{i}\times \frac{1}{L}\sum_{i=1}^{L}R_{i}}{\frac{1}{L}\sum_{i=1}^{L}P_{i}+\frac{1}{L}\sum_{i=1}^{L}R_{i}}$$

where: *L* is 12, and *P_i_* and *R_i_* are the precision and recall of the *i*th locomotion classification, respectively.

The *macro-F1* score results are shown in Table 8. LightGBM had the best generalization classification performance among the five algorithms.

Table 9 illustrates the calculation time of the five models, in which N_S_ is the number of samples to be classified. In the present study, the calculation was performed using the following: I7-8550U, 1.8 GHz CPU and 16G RAM, and Pycharm IDE. It can be observed that the SVM classifier had a slightly longer computational time, when compared to the LDA classifier, and that the computational complexity for the weighted majority voting method was larger, when compared to the majority voting method.

LightGBM had an average of 2.4 s for training computational time, this far outweighs the other four methods. However, it obtains a short average prediction time (15 ms). Hence, LightGBM is still a more ideal model.

## 4. Discussion

In this paper, locomotion mode recognition for walking on three terrains based on sEMG of lower limb and back muscles was presented. Previous studies have used classification algorithm to separate several phases of gait on a specific terrain or differentiate terrains on gait cycles [4,5,9,10]. To the authors’ knowledge, this is the first study in which gait phase events on more than one terrain have been classified to provide both gait phase and terrain information.

Researchers have investigated the sEMG of the lower limbs during walking to identify lower limbs motion [41]. A previous study has detect the knee motion intention from sEMG of erector spinalis muscle with a high accuracy (>95%) [42]. Considered the map of muscle relationships of the locomotion system, erector spinalis of back is active around both heel strike and toe-off during walking. This paper chooses this back muscle and other four lower limb muscles for walking experiments. The usage of multiple EMG electrodes increases data dimensions and computation time. Thus, combinations of fewer EMG channels to obtain an optimal electrodes array will be studied in the future.

Feature extraction and selection is an important step in locomotion mode pattern recognition. Previous studies have shown sEMG features based on the time domain information and the wavelet transform could achieved considerable accuracy for classifying hand movements and gait events [6,7,11,21]. This paper uses statistic criteria to evaluate the 35 EMG features of time domain, frequency domain and time-frequency domain. Due to the lower complexity of computation and better distinguishing ability, when compared to Davies-Bouldin index and scattering index, the rate between the Euclidean distance and standard deviation is a more effective indicator to evaluate the features. Three time-domain features and three Daubechies wavelet transform based time-frequency domain features which have better class separability and stronger robustness are chosen as inputs of LDA or SVM classifiers.

Classification results on individual classifier and multiple classifier fusion both show LDA performs significantly weaker than SVM in most cases. This result is consistent with that in previous researches [43]. Although the improvement in classification accuracy for most locomotion can be observed in the majority voting fusion and weighted majority voting fusion methods, when compared to the average performance of individual classifier, multiple classifiers methods exhibit poor performance in the locomotion that individual classifier do not accurately identify. The reason is that individual classifier has similar low classification performance, voting fusion yields a worse accuracy. It can also explain why the majority voting fusion method and weighted majority voting fusion method are close in terms of classification performance. Continuous efforts on feature extraction and selection would be required to obtain features which can guarantee both good and independence classification performance under various locomotion. Then better classification performance on voting fusion methods can be expected. In addition, feature based on other signals (accelerometer signals or angular velocity signals) can be used to train a independence classifier, improving classification accuracy of some locomotion which can not be better differentiated by sEMG feature based classifier.

LightGBM is superior to traditional fusion methods in locomotion mode recognition. Improvement in the classification accuracy of locomotion on which individual classifier and multiple classifier fusion exhibit poor performance can be acquired in LightGBM. LightGBM had a better performance in the worst case scenario. Although its training computational time outweighs the other methods, LightGBM obtains a short average prediction time. Futhermore, the macro-F1 score results show LightGBM had the best generalization classification performance among the five algorithms.

This research will be go on to advance in the following parts: In terms of electrodes, different neuromuscular control information will be studied to obtain an optimal sEMG electrodes array. In terms of signal source, accelerometer signals or angular velocity signals will be used to fuse with sEMG. In addition, EEG-EMG coherence between the motor cortex and lower leg muscle which can reveal engagement of motor cortex during locomotion tasks is expected as an effective metrics for gait identification. But there is no acknowledged way to remove motion artifacts in EEG during walking. In terms of terrains, application scenarios will consider other terrains of daily living, such as ramp, obstacle and so on. Finally, the research will demonstrate the accuracy of real-time classification.

## 5. Conclusions

The present study analyzed the performance of five ensemble learning algorithms in recognizing the gait phase events on three terrains that exist in daily life. The time domain, frequency domain and time-frequency features based on the sEMG of the five muscles of the lower limb and back were extracted. Then, three feature selection methods were used to evaluate the class separability and computation complexity of the features. After the quantitative analysis of features based on the rate between the Euclidean distance and standard deviation (RES) feature selection method, the mean absolute value (MAV), waveform length (WL), root mean square (RMS), absolute mean value of Db_1_ wavelet decomposition detailed coefficient on level 2 (Db_1_-cD_2_-MAV), waveform length of the DB1 wavelet decomposition detailed coefficient on level 1 (Db_1_-cD_1_-WL), and waveform length of the Db_1_ wavelet decomposition detailed coefficient on level 2 (Db_1_-cD_2_-WL) were chosen as the optimal feature set. Furthermore, the accuracy rate, macro-F1 score, and calculation time of the five ensemble learning algorithms were synthetically considered. On one hand, the LightGBM algorithm was proven to have a better average accuracy rate and generalization performance, when compared to the traditional voting fusion decision making algorithm in locomotion mode recognition. On the other hand, LightGBM obtains a comparable prediction time to other algorithms, although this has a larger training computational time. Overall, LightGBM is a more superior model to effectively improve the recognition rate of locomotion mode.

## Figures and Tables

**Table 1:** Gait space of muscles of the lower extremities.

Muscle Space	Muscles
Biphasic space	Sartorius, Erector spinalis, Adductor longus
Propulsion space	Soleus, Lateral gastrocnemius, Medial gastrocnemius, Peroneus longus
Heel strike space	Tibialis anterior, Extensor digitorum longus, Medial hamstrings, Lateral hamstrings
Loading response space	Vastus lateralis, Rectus femoris, Gluteus medius

**Table 2:** Overall classification accuracy of individual sEMG features, LDA classifier.

Subject	LDA					
	MAV	WL	RMS	Db1-cD2-MAV	Db1-cD1-WL	Db1-cD2-WL
S1	91.82%	89.09%	86.14%	88.86%	84.32%	85.45%
S2	81.85%	88.89%	81.30%	84.44%	87.96%	82.96%
S3	86.3%	84.09%	83.26%	82.02%	83.68%	78.93%
S4	79.33%	80.31%	77.95%	77.95%	79.92%	74.21%

**Table 3:** Overall classification accuracy of individual sEMG features, SVM classifier.

Subject	SVM					
	MAV	WL	RMS	Db1-cD2-MAV	Db1-cD1-WL	Db1-cD2-WL
S1	91.59%	91.36%	90.23%	88.64%	87.27%	85.45%
S3	84.26%	89.26%	85%	86.3%	88.52%	86.3%
S4	88.64%	85.74%	86.78%	85.33%	82.85%	78.51%
S5	81.1%	82.68%	80.91%	82.09%	83.27%	80.71%

**Table 4:** Detailed classification performance for the five ensemble learning methods; Subject #1.

Subject	Terrain	Phase	Classifier				
			MV-LDA	MV-SVM	WV-LDA	WV-SVM	LightGBM
S1	Ground	Post_HC	97.37%	94.74%	97.37%	94.74%	97.37%
		Pre-TO	97.37%	100%	97.37%	100%	84.21%
		Post_TO	97.37%	92.11%	94.74%	92.11%	97.37%
		Pre-HC	97.37%	100%	97.37%	100%	100%
	Stairs Ascent	Post_HC	88.89%	91.67%	88.89%	88.89%	83.33%
		Pre-TO	86.11%	88.89%	88.89%	86.11%	94.44%
		Post_TO	97.22%	97.22%	100%	100%	94.44%
		Pre-HC	47.22%	72.22%	44.44%	72.22%	75%
	Stairs Descent	Post_HC	97.22%	100%	97.22%	100%	97.22%
		Pre-TO	94.44%	100%	94.44%	100%	97.22%
		Post_TO	100%	100%	100%	100%	97.22%
		Pre-HC	75%	69.44%	75%	72.22%	88.89%
	Overall		89.77%	92.27%	89.77%	92.27%	92.73%

**Table 5:** Detailed classification performance for the five ensemble learning methods; Subject #2.

Subject	Terrain	Phase	Classifier				
			MV-LDA	MV-SVM	WV-LDA	WV-SVM	LightGBM
S2	Ground	Post_HC	86.11%	91.67%	86.11%	90.28%	93.06%
		Pre-TO	94.44%	100%	94.44%	100%	98.61%
		Post_TO	98.61%	100%	98.61%	100%	98.61%
		Pre-HC	81.94%	90.28%	80.56%	90.28%	94.44%
	Stairs Ascent	Post_HC	70.97%	70.97%	70.97%	67.74%	77.42%
		Pre-TO	87.10%	74.19%	83.87%	74.19%	87.10%
		Post_TO	100%	100%	100%	100%	100%
		Pre-HC	80.65%	70.97%	80.65%	70.98%	87.10%
	Stairs Descent	Post_HC	81.25%	81.25%	84.38%	90.63%	90.63%
		Pre-TO	93.75%	100%	90.63%	96.88%	93.75%
		Post_TO	100%	90.63%	100%	90.63%	93.75%
		Pre-HC	59.38%	59.38%	59.38%	59.38%	84.36%
	Overall		87.41%	88.70%	87.04%	88.70%	92.96%

**Table 6:** Detailed classification performance for the five ensemble learning methods; Subject #3.

Subject	Terrain	Phase	Classifier				
			MV-LDA	MV-SVM	WV-LDA	WV-SVM	LightGBM
S3	Ground	Post_HC	86.67%	90%	76.67%	86.66%	90%
		Pre-TO	100%	100%	96.67%	100%	100%
		Post_TO	90%	100%	90%	100%	96.67%
		Pre-HC	86.67%	80%	76.67%	83.33%	86.67%
	Stairs Ascent	Post_HC	82.69%	90.38%	82.69%	88.46%	92.31%
		Pre-TO	76.92%	88.46%	75%	88.46%	94.23%
		Post_TO	100%	100%	100%	100%	98.07%
		Pre-HC	69.23%	76.92%	73.08%	76.92%	92.31%
	Stairs Descent	Post_HC	92.31%	97.44%	92.31%	100%	97.44%
		Pre-TO	74.36%	74.36%	79.48%	76.92%	89.74%
		Post_TO	97.44%	92.31%	97.44%	92.31%	94.88%
		Pre-HC	87.18%	82.05%	87.18%	82.05%	92.31%
	Overall		86.16%	89.05%	85.33%	89.26%	93.8%

**Table 7:** Detailed classification performance for the five ensemble learning methods; Subject #4.

Subject	Terrain	Phase	Classifier				
			MV-LDA	MV-SVM	WV-LDA	WV-SVM	LightGBM
S4	Ground	Post_HC	86.67%	84.44%	86.67%	84.44%	97.37%
		Pre-TO	100%	100%	100%	100%	84.21%
		Post_TO	93.33%	93.33%	93.33%	91.11%	97.36%
		Pre-HC	55.56%	84.44%	55.56%	84.44%	100%
	Stairs Ascent	Post_HC	66.67%	64.58%	56.25%	52.08%	83.33%
		Pre-TO	85.42%	79.12%	81.25%	77.08%	94.44%
		Post_TO	95.83%	93.75%	95.83%	95.83%	94.44%
		Pre-HC	66.67%	68.75%	68.75%	72.91%	75%
	Stairs Descent	Post_HC	73.53%	85.29%	73.53%	88.24%	100%
		Pre-TO	94.12%	91.18%	97.06%	91.12%	97.22%
		Post_TO	100%	100%	100%	100%	100%
		Pre-HC	55.88%	73.53%	55.88%	76.47%	88.89%
	Overall		81.1%	84.45%	80.12%	83.86%	92.73%

**Table 8:** The *macro-F1* score results for the five ensemble learning methods.

Subject	*macro-F1*				
	MV-LDA	MV-SVM	WV-LDA	WV-SVM	LightGBM
S1	0.896	0.923	0.896	0.923	0.928
S2	0.867	0.867	0.86	0.867	0.918
S3	0.869	0.892	0.858	0.895	0.94
S4	0.818	0.849	0.807	0.844	0.928
Average	0.863	0.883	0.855	0.882	0.929

**Table 9:** Calculation time.

Subject	Ns	MV-LDA		MV-SVM		WV-LDA		WV-SVM		LightGBM	
		T_Training_	T_Prediction_	T_Training_	T_Prediction_	T_Training_	T_Prediction_	T_Training_	T_Prediction_	T_Training_	T_Prediction_
S1	440	0.043	0.014	0.1	0.016	0.113	0.015	0.231	0.02	1.999	0.012
S2	540	0.075	0.016	0.13	0.022	0.294	0.025	0.132	0.018	3.216	0.02
S3	484	0.042	0.016	0.119	0.023	0.115	0.017	0.276	0.026	2.501	0.014
S4	508	0.062	0.018	0.124	0.023	0.113	0.016	0.274	0.025	2.071	0.013

## Data Availability

Not applicable.
